# Humor

**Published:** 1888-12

**Authors:** 


### HUMOR.

(*Medical and Surgical Reporter, Philadelphia.*)

To be Developed Later.—"So your uncle is dead, Charley?" "Yes, died yesterday." "He was a very eccentric old fellow. Do you think he was altogether right in his head?" "Well—er—I couldn't say, you know, until the will is read."

The Coachman's Diagnosis.—Gentleman, to the doctor's driver: "Tell me, how is the Councillor getting along?" Driver: "Oh, we've got him well; but the General is doing badly; we're having a great deal of trouble with him."—*Fliegende Blätter.*

Something Peculiar.—A. "My cousin is a queer fellow. I never know what he will do next; but it is always something peculiar." B. "For example?" A. "Well, just think, in the summer of 1888, the fellow got himself sun-struck?"—*Fliegende Blätter.*

Loyal to the Last.—Husband (returning from the mountains)—"Are you chilly, my dear?" Wife—"I'm nearly frozen, John. Who was that gentleman in the straw hat and linen duster who just drove by?" Husband—"The landlord of the hotel below here."

Forgot About It.—Brown—"Have you seen Robinson recently, Dumley? I hear he has been sick." Dumley—"Yes, I saw him this morning." Brown—"How is he?" Dumley—"By thunder, I forgot to ask him. I just said, 'How are you, old man?' and passed on."—*Time.*

A Remarkable Remedy.—"Doctor, I can't take this medicine; it tastes horrible!" "Oh, never mind; I have the most extraordinary results from this drug. I prescribe it for all my patients. None of them take it; and they all get well!"—*Fliegende Blätter.*

For Value Received.—A correspondent writes to the *Cincinnati Lancet-Clinic*, October 6, 1888, that he on one occasion went to a meeting in which the pastor called on the old family physician of the neighborhood to offer prayer. The latter arose, and after an invocation of several minutes' duration, ended his petition with this remarkable appeal: "And now, oh Lord, please be adoin' better by us in the future than you've been adoin' in the past, and then to you will be the power, glory, and everlastin' singin.' Amen."

